# Inference in epidemiological agent-based models using ensemble-based data assimilation

**DOI:** 10.1371/journal.pone.0264892

**Published:** 2022-03-04

**Authors:** Tadeo Javier Cocucci, Manuel Pulido, Juan Pablo Aparicio, Juan Ruíz, Mario Ignacio Simoy, Santiago Rosa

**Affiliations:** 1 FaMAF, Universidad Nacional de Córdoba, Córdoba, Córdoba, Argentina; 2 FaCENA, Universidad Nacional del Nordeste, Corrientes, Corrientes, Argentina; 3 IFAECI, CONICET, Corrientes, Corrientes, Argentina; 4 IMIT, CONICET, Corrientes, Corrientes, Argentina; 5 INENCO, CONICET, Universidad Nacional de Salta, Salta, Salta, Argentina; 6 Simon A. Levin Mathematical, Computational and Modeling Sciences Center, Arizona State University, Tempe, Arizona, United States of America; 7 CIMA, CONICET, Universidad de Buenos Aires, Ciudad Autónoma de Buenos Aires, Buenos Aires, Argentina; 8 Sciences of the Atmosphere and the Oceans Department, FCEN, Universidad de Buenos Aires, Ciudad Autónoma de Buenos Aires, Buenos Aires, Argentina; 9 Instituto Multidisciplinario sobre Ecosistemas y Desarrollo Sustentable, Universidad Nacional del Centro de la Provincia de Buenos Aires, Tandil, Argentina; Vilnius University, LITHUANIA

## Abstract

To represent the complex individual interactions in the dynamics of disease spread informed by data, the coupling of an epidemiological agent-based model with the ensemble Kalman filter is proposed. The statistical inference of the propagation of a disease by means of ensemble-based data assimilation systems has been studied in previous works. The models used are mostly compartmental models representing the mean field evolution through ordinary differential equations. These techniques allow to monitor the propagation of the infections from data and to estimate several parameters of epidemiological interest. However, there are many important features which are based on the individual interactions that cannot be represented in the mean field equations, such as social network and bubbles, contact tracing, isolating individuals in risk, and social network-based distancing strategies. Agent-based models can describe contact networks at an individual level, including demographic attributes such as age, neighborhood, household, workplaces, schools, entertainment places, among others. Nevertheless, these models have several unknown parameters which are thus difficult to prescribe. In this work, we propose the use of ensemble-based data assimilation techniques to calibrate an agent-based model using daily epidemiological data. This raises the challenge of having to adapt the agent populations to incorporate the information provided by the coarse-grained data. To do this, two stochastic strategies to correct the model predictions are developed. The ensemble Kalman filter with perturbed observations is used for the joint estimation of the state and some key epidemiological parameters. We conduct experiments with an agent based-model designed for COVID-19 and assess the proposed methodology on synthetic data and on COVID-19 daily reports from Ciudad Autónoma de Buenos Aires, Argentina.

## Introduction

Prediction models usually represent a system through a set of differential equations that govern the evolution of the system through continuous variables. Agent-based models (ABMs) rely on a different paradigm. They explicitly represent the characteristics and behavior of interacting autonomous individuals –usually referred to as agents– and use them to simulate scenarios which serve as a modelization of complex systems [1]. Even simple interactions rules may lead to self-organization and emerging collective behavior [2]. Therefore, ABMs follow a bottom-up approach in describing the dynamics of the system. They are useful to model the dynamics of epidemiological, ecological, economical, and social systems [3–6].

From a computational point of view, each agent consists of a collection of data attributes that describes its state. The behavior and interaction of agents are governed via autonomous decisions and/or probabilistic rules which eventually modify the agent’s status. ABMs can be interpreted from the perspective of object oriented programming (indeed, many implementations follow this paradigm) where each agent is an instance of a class with certain attributes. These attributes can be real numbers (e.g., space coordinates) or categorical variables (e.g., epidemiological state, social class), among other possible data types. The possibility of describing complex behavior through a potentially simple set of rules has fostered the popularity of ABMs in recent years. The computational cost is not negligible for systems with a large number of agents, however nowadays this is not a strong limitation due to the increase of computational power.

By describing the epidemiological state of individuals and their network of social contacts, epidemiological ABMs are suitable to represent quite realistically the evolution and spread of infections [7]. The use of ABMs to represent infectious disease dynamics is promising because infections are indeed produced by contacts between people, and ABMs allow to model at this (micro)scale. In fact, it is quite straightforward to represent what is known about human interactions, through the interaction of agents. To model COVID-19 dynamics a number of ABMs have been developed which, among other features, include age structure and represent a social network that includes schools, houses, workplaces, etc. to provide realistic mixing (see, for example, [8–10]). Considering this social structure, the assessment of the effects of non-pharmaceutical interventions, such as confinement measures, closing of schools, social gathering limitations, can be captured and simulated with ABMs. A strong signature of the last decade has been the increase of devices (GPS, cameras, digitalized reports, commercial records) that can collect information at the human level. The potential of ABMs to model the complex interactions between individuals and to foster the use of anonymized individual-based information is huge. In this line, [11] use mobility and demographic data to construct the contact network and household distribution for a COVID-19 ABM which is used to assess the effect of non-pharmaceutical interventions.

One of the main limitations of ABMs is the need for setting multiple simulation parameters. Recently, there were efforts to develop inference techniques to constrain ABM parameters through available observations. These are mainly focused on obtaining a proxy for the likelihood. In [12], a variety of methods are proposed to calibrate ABMs, for instance, using Approximate Bayesian Computation alongside Markov chain Monte Carlo or approximating the likelihood using an emulator for the ABM.

The ensemble Kalman filter (EnKF) is a data assimilation (DA) technique suitable to conduct sequential Bayesian inference in noisy partially observed systems. Both, model predictions and observations, are assumed to have Gaussian uncertainties. The Gaussian assumption represents its main weakness and, at the same time, its main strength. Non-Gaussian uncertainties may result in a sub-optimal performance of the filter. On the other hand, a relatively small number of sample points –called ensemble members or particles– may suffice for high dimensional state spaces. In particular, the correlation between variables can be well captured because of the Gaussian assumption. Observations of the system’s state variables are often incomplete (not all state variables are observed) and indirect (observed variables are a function of the state variables). By considering the correlations between state variables, the EnKF can use the observations to improve the estimates of every state variable, even those which are not observed [13]. Furthermore, unknown parameters of the model can be treated as unobserved variables and included in the state. Therefore, if there is enough correlation between the parameters and the observed variables, the EnKF is able to produce estimates of the unknown model parameters. This procedure, called state augmentation, is quite straightforward to implement [14, 15].

Although the EnKF has been originally developed for numerical weather prediction [16, 17], its field of application has broadened over time. In particular, some works have applied the EnKF in epidemiological systems. In [18, 19] the ensemble adjustment Kalman filter (EAKF) is used to forecast the times at which the peaks for influenza outbreaks were reached. More recently, there are applications of the EnKF to infer COVID-19 transmission dynamics. In [20] iterated filtering using the EAKF is applied to estimate undocumented cases in China. Iterated filtering was earlier introduced by Ionides et al [21] based on particle filters with state augmentation, to provide maximum likelihood estimates of model parameters and used it to study cholera dynamics. In [22] the use of an ensemble Kalman smoother with multiple data assimilation (ESMDA) is proposed to estimate the parameters, mainly focused on the effective reproduction number, of a COVID-19 compartmental model. In [23] an EnKF is used to study the impact of vaccination for COVID-19 using data from Saudi Arabia. All of these contributions use compartmental epidemiological models represented through differential equations.

The application of ensemble-based DA concepts to combine ABMs with data raises several challenges. A pioneer work in this direction combining the ensemble Kalman filter with an ABM was conducted by Ward et al [24]. In that work, an ABM, combined with an ensemble Kalman filter to assimilate data from footfall cameras, was used to study pedestrian behavior. They obtained satisfactory results, but the authors also note the challenges presented by parameter sensitivity and the need for parallelization when using large models. In ABMs, the model state is defined at a micro-scale as the set of current values of the attributes for each agent. The model provides the evolution of the attributes of each agent which are modified in time as a result of the interactions between them. For decision making, the individual state of each agent is not necessarily of interest and what is relevant are aggregated quantities and/or an anonymized group of individuals representing a certain class of attributes. These variables summarize information on the population as a whole. Even when the hidden state is at the micro-scale, and the inference’s goal is to represent this microscopic state as closely as possible, observations are usually of the macroscopic scale so that the microscopic state is not well constrained by observations. We propose to conduct DA in the space of these aggregated variables. The mapping of the micro into the macro state is straightforward by aggregating the variables of interest for assimilation. But this is not the case for the inverse mapping. The aggregated variables are not necessarily matched with a single microscopic state. In fact, it is likely that many microscopic states of the agent population yield the same aggregated variables. This issue needs to be addressed in order to produce realistic forecasts. Two methodologies are proposed and evaluated in this work to produce the mapping between the macroscopic state to the microscopic state.

In this work, we provide and evaluate a general framework to use ensemble-based DA on epidemiological ABMs. In the Methods section we introduce an ABM with spatial and social structure designed for COVID-19 spread. We also give a general framework of DA and, in particular, ensemble-based DA. Then we discuss a general methodology to apply ensemble-based DA to ABMs. Two implementations of the DA-ABM coupling are presented for the specific case of our epidemiological ABM. These coupling methodologies are used to assimilate observations within the ensemble Kalman filter. In the Experiment and results section, the system is assessed in experiments using synthetic observations, and then we use it on real COVID-19 data from Ciudad Autónoma de Buenos Aires (CABA), Argentina.

## Methods

### Epidemiological modeling

To model disease dynamics, the seminal work of Kermack et al [25] represents a population divided in compartments. The basic *SIR* model considers three compartments related to the disease status of the individuals: Susceptible, Infected and Recovered. This has become common practice in epidemiological modeling. Dividing the population in subpopulations, under the assumption of homogeneity in each of them, allows for a small number of variables to summarize the state of the system. These indicate how many individuals are in each compartment. The standard SIR model can be modified and many different compartment configurations can be set up in order to better represent the main characteristics of different diseases. Analyzing the flow between compartments provides a general understanding of the disease dynamics. These models only keep track of the macro-state variables –the number of individuals in each compartment– but they do not model the individuals themselves. Compartmental models are commonly represented by a system of differential equations which can be integrated to get the time evolution of the disease dynamics.

A variety of compartmental models have been used to model COVID-19 which include different traits and complexities [26]. They have proven useful to estimate epidemiological parameters, predict trends, and evaluate control measures [27]. Metapopulation compartmental models may represent populations with age structure (e.g., [22]), geolocalization (e.g., [28]), and may include stochastic transmission. Furthermore, the representation through differential equations of the system allows to formally analyze the dynamical system’s behavior [29]. On the other hand, the complex interactions between individuals are averaged out in this type of models because of the assumptions of mean field interactions and well-mixed populations.

ABMs aim to model people’s behavior, represented by agents, explicitly. Each individual of the population is labeled with an epidemiological status (here referred to as epidemiological class or category). Infections are caused by the interactions between agents, and labels can be changed accordingly. Usually, what is of interest is not the particular outcome of the simulation of an individual but rather the resulting state of the system as a whole. Simple interaction rules can yield complex global behavior. In this case, the total number of agents at each category at a given time gives the aggregated representation of the agent-based system. The resulting aggregated variables are not modeled with differential equations: the state of each subpopulation emerges from the individual-based level. ABMs also allow the straightforward introduction of relevant features and complexities to the model. For example, waning immunity or health measures such as lockdowns, contact tracing, or vaccination effects can be implemented in a very explicit fashion [30]. In ABMs instead of assuming that the population is well-mixed, interactions are modeled in an individual basis so that complex networks can be used to give richer agent interactions. These features may be very difficult to represent mathematically with differential equations. The bottom-up approach of ABMs gives them great expressiveness and flexibility with little modeling efforts.

#### Agent-based model details

The ABM we developed was designed to model disease dynamics, in particular for COVID-19 spread. Individuals are characterized mainly by three properties: disease status, house, and neighborhood. However, the model framework easily allows to include additional properties such as age structure, occupation, or social stratum. We name our model epiABM and in this section we describe how it operates.

**Fig 1:**
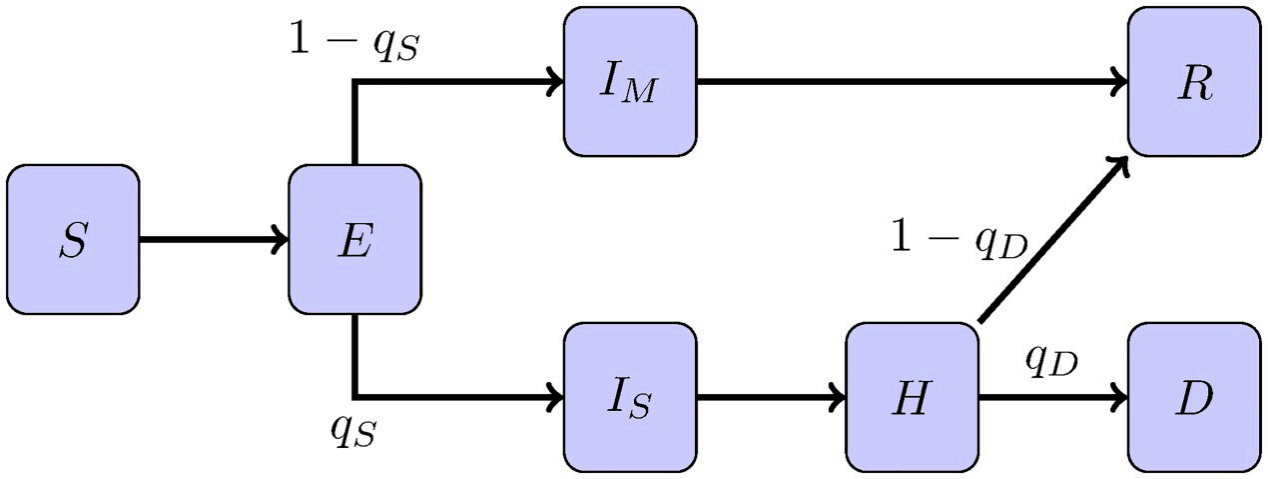
Diagram for the epidemiological classes in epiABM. Susceptible (*S*), Exposed (*E*), Mild infected (*I*_*M*_), Severe Infected (*I*_*S*_), Hospitalized (*H*), Recovered (*R*) and Deceased (*D*).

The disease status of each individual is described by one of seven categories. Namely, we have the susceptible individuals (*S*) for agents which can get infected, the exposed class (*E*) for those that have been infected but are not yet infectious. The infectious individuals are divided in two groups. The mildly infected class (*I*_*M*_) is intended for individuals that develop non-hospitalizable form of the disease, including asymptomatic ones, and are expected to recover. The severely infected (*I*_*S*_) are individuals that will require hospitalization. The hospitalized ones (*H*) can recover or die. Finally, we have the recovered (*R*) and the deceased individuals (*D*). We assume that recovered individuals develop immunity for the duration of the simulation, but note that this would be unrealistic for longer-term simulations. Hereinafter, we use these symbols (*S*, *E*, *I*_*M*_, *I*_*S*_, *H*, *R*, *D*) to alternatively denote the label of the health status of the individual or the population size of the class. A diagram of the flow between the epidemiological classes is shown in Fig 1. Note that we are referring to (*S*, *E*, *I*_*M*_, *I*_*S*_, *H*, *R*, *D*) to the *epidemiological classes of the individuals*. In compartmental models, these symbols represent variables which are modeled via a set of differential equations with mean-field interactions terms.

At every time step, which is considered a day by default, each agent has a number of contacts sampled from a Poisson distribution with parameter λ. Susceptible agents may become exposed as a result of a contact with an infectious agent: in a contact between agents, if one of them is infectious and the other susceptible, there is a chance that the latter becomes infected too. The time spent in each infected class (*E*, *I*_*M*_, *I*_*S*_, *H*) is considered as a Gamma distribution following [31].

The time *τ*_*c*_ spent by an agent in class *c* ∈ {*E*, *I*_*M*_, *I*_*S*_, *H*} is sampled from a Gamma distribution, i.e. *τ*_*c*_ ∼ Γ(*k*_*c*_, *θ*_*c*_) where *k*_*c*_ and *θ*_*c*_ are the shape and scale parameters for Gamma distributions. The mean and variance are given by *μ*_*c*_ = *k*_*c*_*θ*_*c*_ and $\sigma_{c}^{2}=k_{c}\theta_{c}^{2}$ respectively. When this time *τ*_*c*_ is spent, the agent will move on to the next epidemiological status. When an agent exits the exposed category, it has a chance of *q*_*S*_ of developing a severe sickness and a probability *q*_*M*_ = 1 − *q*_*S*_ of having mild symptoms. In a similar fashion, hospitalized agents have a *q*_*D*_ chance to die and a *q*_*R*_ = 1 − *q*_*D*_ chance to recover.

In addition to the structure given by the health status of the agents, we introduce geographic and demographic information. We consider a city divided into *N*_*loc*_ neighborhoods. Each agent lives in a house in a certain neighborhood. The house can hold a single agent or it can be shared with others. Contacts are categorized into two cases: domestic and casual contacts. Each of the daily contacts of an agent has a chance *q*_*C*_ of being casual and 1 − *q*_*C*_ of being domestic. Domestic contacts are between members of the same household and casual contacts are potentially with any other agent. The probability of infection for a domestic contact is *β*_*d*_ and it is *β*_*c*_ for casual contacts. The expected global probability of infection is then *β* = *β*_*c*_*q*_*C*_ + *β*_*d*_(1 − *q*_*C*_). The probability of a casual contact of an individual of neighborhood *j* with an individual of the neighborhood *i* is *C*_*ij*_. This results in a *N*_*loc*_ × *N*_*loc*_ matrix which we call contact matrix. The diagonal elements correspond to the probability of a casual contact to be between agents within the same neighborhood. The off-diagonal terms are related to contacts of individuals that visit other neighborhoods. This contact matrix encodes agent mobility between neighborhoods which in turn is related to work and social activities. This matrix may be designed to model different characteristics of the social and geographic structure. For example it would be expectable that agents move more frequently around their own neighborhoods and this would mean that the diagonal values are larger. Off-diagonal terms representing inter-neighborhoods contacts would then be smaller. A more visited neighborhood, such as the city center is represented with larger off-diagonal terms. This contact matrix is assumed fixed in this work, but in order to model this matrix in a more realistic manner, mobility data from smartphones could be used to fit a time-varying contact matrix which can track changes in mobility.

Further structure and classes in the population may be incorporated. For example, age, social profile, occupation or school attendance which can be helpful to represent phenomena such as superspreader events. This model could also be adapted to better represent the latest developments regarding the disease, such as vaccinations or the new virus variants. New fields can be added to the agent’s inner structure to state if it is vaccinated or not (or even how many doses it received). Also, another attribute for the infected agents may indicate which virus variant it is hosting. Further detail may be added to contacts by modeling through the contact matrix, the effect on mobility changes, as lockdowns begin to be lifted and schools, workplaces and social gathering venues begin to reopen. We have restricted the contact structure to its minimal expression but keeping house granularity since the main aim of this article is to evaluate the inference technique with widely available data. Because of the effect of higher infection rates and non-viability of distance measures within houses, we considered house granularity was one of the essential individually-based structures to evaluate in the data assimilation system.

In the ABM we developed, and in general, each agent is labeled with a single epidemiological class. However, ABMs cannot be described with a system of ordinary differential equations (ODEs) as in compartmental models. For cases with a large amount of agents, the resulting aggregated variables of the ABM can smooth out the effect of stochastic components (for example, the Gamma-distributed time of residence of an agent in an epidemiological class) and yield results which may be reproducible with a compartmental ODE model. However, ABMs do not have an obvious ODE counterpart. It is not clear how some features of the ABM, such as the behavior which emerges from having agents residing in houses with different probabilities for domestic or casual contacts, should be represented through ODEs. Using ABMs allows to include such features and complexities at a microscopic-level which have consequences at a larger scale that are hard to model and thus hard to reproduce with differential equations. It is worth noting that ABMs are usually computationally demanding to simulate from. Because of this, in the case that a surrogate ODE emulator of an ABM is available, it could be used to infer some model parameters at a low computational cost (as proposed in Hooten et al [12]).

#### Default parameterization

Table 1 summarizes the default parameters used in the experiments. We choose parameters that are representative of the early stage of the COVID-19 pandemic. We do not aim for medical accuracy since the goal of the experiments is to evaluate the methodology in different scenarios. A mean incubation period of 4 days is reported in [32], which is consistent with our Gamma parameterization, *μ*_*E*_ = *k*_*E*_*θ*_*E*_ = 4 days. The mean infectious period for mild infections is chosen to be $\mu_{I_{M}}=k_{I_{M}}\theta_{I_{M}}=8\;\text{days}$ and values around this are used in other models [33, 34] and reported in [35]. We use $\mu_{I_{S}}=k_{I_{S}}\theta_{I_{S}}=8\;\text{days}$ as the mean time between severe illness and hospitalization which is in the range reported in [36]. The mean time agents spend hospitalized is chosen to be *μ*_*H*_ = *k*_*H*_*θ*_*H*_ = 8.1 days which is compatible with the results in [37]. We set the probability of severe symptoms *q*_*S*_ of 10% and of death of the severely infected *q*_*D*_ of 40% which yields a case fatality rate of 4%. These values are in line with the early stages of the pandemic: in mainland China, up to February 2020, a 3.67% case fatality ratio was reported [38]. Similar values were found in Argentina in early-stage experiments [22]. After vaccination and with the mutations of the virus the fatality ratio has diminished substantially from these values. Since we have different configurations of λ in every experiment, we do not provide a default value. The expected number of infections that an infected agent produces in a totally susceptible population is λ*β*. With our choices of λ alongside the defaults for *β*_*c*_, *β*_*d*_ and *q*_*C*_ we get that this quantity is similar to that yielded by the defaults used in [8]. The default distribution of houses according to the number of inhabitants is given by a vector of probabilities *p*_*H*_ for which the probability of a house to have *i* members is given by the i-th entry of this vector. Houses with more than 5 individuals are not included in the model, i.e., we assume *p*_*H*_ is of dimension 5. As a default we use *p*_*H*_ = (0.36, 0.27, 0.16, 0.13, 0.08) in both synthetic and realistic experiments, except in the model error experiment (for which *p*_*H*_ specified in its description). These are reference values taken from a demographic survey in CABA, Argentina (specifically Encuesta Anual de Hogares called EAH 2019).

**Table 1:** Default parameters for epiABM.

Parameter	Description	Value
*β*_*d*_	Infection probability in domestic contact	0.8
*β*_*c*_	Infection probability in casual contact	0.16
*q*_*D*_	Death probability for hospitalized individuals	0.4
*q*_*S*_	Probability of severe symptoms development	0.1
*q*_*C*_	Probability of a contact to be casual	0.5
*k*_*E*_	Shape parameter of Gamma distribution for time at *E*	1.78
*θ*_*E*_	Scale parameter of Gamma distribution for time at *E*	2.25
$k_{I_{M}}$	Shape parameter of Gamma distribution for time at *I*_*M*_	7.11
$\theta_{I_{M}}$	Scale parameter of Gamma distribution for time at *I*_*M*_	1.13
$k_{I_{S}}$	Shape parameter of Gamma distribution for time at *I*_*S*_	4.0
$\theta_{I_{S}}$	Scale parameter of Gamma distribution for time at *I*_*S*_	1.0
*k*_*H*_	Shape parameter of Gamma distribution for time at *H*	9.0
*θ*_*H*_	Scale parameter of Gamma distribution for time at *H*	0.9

### Data assimilation framework

In the standard DA framework, we have noisy partial observations $y_{1},\ldots ,y_{T}\in R^{N_{y}}$ of a time evolving process of latent (or hidden) state variables $x_{0},x_{1},\ldots ,x_{T}\in R^{N_{x}}$ where the subindices represent discrete times. The initial condition, **x**_0_, is considered to follow a given distribution *p*(**x**_0_), and is conventionally considered unobserved. The evolution of the state and observations is governed by the state-space model equations for *t* = 1, …, *T*:

$$\begin{array}{c} x_{t}=M_{t}\left(x_{t-1},\eta_{t}\right), \end{array}$$ (1)

$$\begin{array}{c} y_{t}=H_{t}\left(x_{t},\nu_{t}\right). \end{array}$$ (2)

With this nomenclature, the state variables at time *t* evolve to the following discrete time through a forward model $M_{t}$ which has stochastic components (represented by the random variable ***η**_t_*). The relation between data and state is modeled through the observational map $H_{t}$ and ***ν**_t_* is a random variable which accounts for the observational error. Eqs 1 and 2 represent a hidden Markov model or state-space model. They determine the transition probability *p*(**x**_*t*_|**x**_*t*−1_) and the observational likelihood *p*(**y**_*t*_|**x**_*t*_), respectively [39].

One of the goals of DA is to incorporate the information of observations into the model predictions of the latent state variables. This means that we are interested in the probability of **x** given **y**_1:*t*_ (where **y**_1:*t*_ ≐ {**y**_1_, ⋯, **y**_*t*_}). In particular, we are interested in the filtering posterior distribution *p*(**x**_*t*_|**y**_1:*t*_). This distribution is usually obtained by a two-step iterative procedure:

- Forecasting step: *p*(**x**_*t*_|**y**_1_, …, **y**_*t*−1_) = ∫*p*(**x**_*t*_|**x**_*t*−1_)*p*(**x**_*t*−1_|**y**_1:*t*−1_)*d* **x**_*t*−1_
- Filtering/analysis step: *p*(**x**_*t*_|**y**_1:*t*_) ∝ *p*(**y**_*t*_|**x**_*t*_)*p*(**x**_*t*_|**y**_1:*t*−1_)

This is a recursive framework: the resulting filtered distribution at time *t* − 1 is used to forecast the distribution at time *t*. The forecast is obtained using the forward model $M_{t}$ (forecasting step). In the filtering step, Bayes’ rule is used to combine the forecast distribution, which is the prior distribution, with the observation likelihood to update the forecast distribution into the posterior distribution [40].

#### Ensemble-based DA

The inference approach of DA is probabilistic, so we are interested in the distribution of the state given the observations. If the resulting filtering distribution is Gaussian, then it would be enough to estimate a mean and covariance matrix to represent this distribution. The Kalman filter gives an exact solution when the prior distribution and the observational likelihood function are Gaussian (which in turn results in a Gaussian filtering distribution). This is guaranteed when the operators $M_{t}$ and $H_{t}$ are linear and the stochastic components ***η**_t_* and ***ν**_t_* are additive Gaussian white noise. In this case, the classical Kalman filter produces a sequence of means ${\left\{x_{t}^{a}\right\}}_{t=1}^{T}$ and covariances ${\left\{P_{t}^{a}\right\}}_{t=1}^{T}$, such that $p\left(x_{t}|y_{1:t}\right)∼N\left(x_{t}^{a},P_{t}^{a}\right)$ [41].

For a non-parametric representation of the distributions, Monte Carlo approaches represent the distributions by a sample. Particle based methods use an ensemble of particles (or ensemble members) to keep track of the forecasting and filtering distribution. These distributions are then represented by an ensemble of states. The general procedure for these methods is to evolve each particle forward using the model to get the ensemble representation of the forecasting distribution and then to transform the states of this ensemble into a sample of the filtering distribution using the information of the observation at that time. This general methodology is specified in Algorithm 1. The procedure used to transform the forecasting ensemble into a filtering ensemble yields different sequential ensemble-based methods. One feature of this framework which will be key for the application to ABMs is that the transition model is basically treated as a black box. This is not the case for the standard Kalman filter for which the linear model is needed explicitly in matrix form for the forecasting to filtering distribution transformation. Two important sample-based families of methods which follow Algorithm 1 stand out: EnKFs and particle filters. If the prediction and observational processes in Eqs 1 and 2 are weakly nonlinear, it is possible to assume that Gaussianity is preserved through the model’s time evolution. Thus, the particles in the filtering step may still assumed to follow Gaussian constraints. This derives in what is known as the EnKF. On the other hand, particle filters do not make any assumptions on the likelihood and prior distributions. They produce a filtered sample by applying Bayes’ rule in a fully non-parametric manner [42].

**Algorithm 1**: General forecasting-filtering scheme for ensemble DA

Sample initial particles: ${\left\{x_{0}^{a(j)}\right\}}_{j=1}^{N_{p}}∼p\left(x_{0}\right)$

**for** *t* = 1, …, *T* **do**

  **for** *j* = 1, …, *N*_*e*_ **do**

   $x_{t}^{f(j)}=M_{t}\left(x_{t-1}^{a(j)},\eta_{t-1}\right)$ using $M_{t}$ from Eq (1)

  **end**

  Transform ${\left\{x_{t}^{f(j)}\right\}}_{j=1}^{N_{p}}$ into ${\left\{x_{t}^{a(j)}\right\}}_{j=1}^{N_{p}}$ using **y**_*t*_

**end**

#### Parameter estimation through state augmentation

When the state of the system is partially observed, DA uses the correlation between observed and unobserved variables to propagate the observational information and improve the estimate of variables that are not observed. With this idea in mind, unknown model parameters can also be interpreted as unobserved variables, and if these are correlated with observed variables, the DA system will calibrate the parameters to values consistent with the observations. To do this, the state is augmented with the parameters, so instead of the state vector **x**_*t*_ we use the augmented vector ${\tilde{x}}_{t}≐\left(x_{t},\theta_{t}\right)$ where ***θ**_t_* are the parameters at time *t*. The model operator $M_{t}$ needs to be extended to also operate in the parameter space and account for the evolution of ***θ**_t_*. A common assumption is to consider that their evolution follows a random walk

$$\begin{array}{c} \theta_{t+1}=\theta_{t}+\epsilon_{t} \end{array}$$

where ***ϵ**_t_* is Gaussian white noise. Also, ***θ***_0_ is considered to be distributed with an a priori distribution based on the range of possible values for ***θ***. A useful feature of this method is that the estimates of ***θ*** can track a parameter that is not constant in time, assuming that the changes are slow [15].

### Data assimilation in ABMs

The state of an ABM at a given time *t* is completely described by the collection of the current values of the attributes for every agent in the ABM. The DA framework previously described cannot be readily applied to this sort of representation because the data attributes which compose agents are not necessarily in a space where DA techniques can be applied. These attributes are computational variables which can be, for example, Boolean or categorical data types. A particle filter may be applicable with categorical variables, but we aim at using the EnKF, which only operates in continuous spaces. Even when agent information may be relevant for shaping the spread of the disease, it is likely that the interest is not on the state of each particular individual but rather on an aggregated global information on the population. We perform the DA process in the space of this aggregated information. Actually, these variables are integer values but can be considered continuous when they are large enough. Observations on the system are more likely to be informative on these aggregated variables and not on specific agents.

Considering the population of agents is a discrete set, we refer to a specific agent with the index *k*. The attributes of the *k*-th agent at time *t* are denoted as *A*_*k*,*t*_. These attributes for each agent define the *micro-state space* $A_{t}={\left\{A_{k,t}\right\}}_{k=1}^{N_{agents}}$ where *N*_*agents*_ is the total number of agents. Given an apriori density of the attributes as a function of the agent classes and the set of model parameters to be calibrated, an ensemble of *N*_*p*_ state members ${\left\{A_{t}^{\left(j\right)}\right\}}_{j=1}^{N_{p}}$ is drawn representing realizations of the apriori density. Naturally, the micro-state forecast density is constructed evolving the agent-based model starting from the drawn initial micro-states.

The process of DA is conducted in a *macro-state space* $x_{t}\in R^{N_{x}}$ composed of aggregated variables and the chosen model parameters. The aggregated (or macroscopic) variables are determined via a map that counts the number of agents in the corresponding class **x**_*t*_ = *ϕ*_*m*→*M*_(*A*_*t*_). In other words, this is a mapping from a micro-structured state (*m*) to a mean field macro-state (*M*). This mapping is not injective and therefore it is not invertible. This means that we have a model to evolve the micro-state *A*_*t*_ into *A*_*t*+1_ and get the resulting **x**_*t*+1_ by performing the aggregating operations. However, once we correct these macro-state variables with DA, we do not have a unique and explicit model to transform them back into the micro-state variables. We denote this macro to micro map as *A*_*t*+1_ = *ϕ*_*M*→*m*_(**x**_*t*+1_). Below, we discuss two different approaches to define the macro to micro map. It is important to note that the EnKF and the particle filter allow the model to be treated as a black box so that the filter is unaware of the mapping.

**Fig 2:**
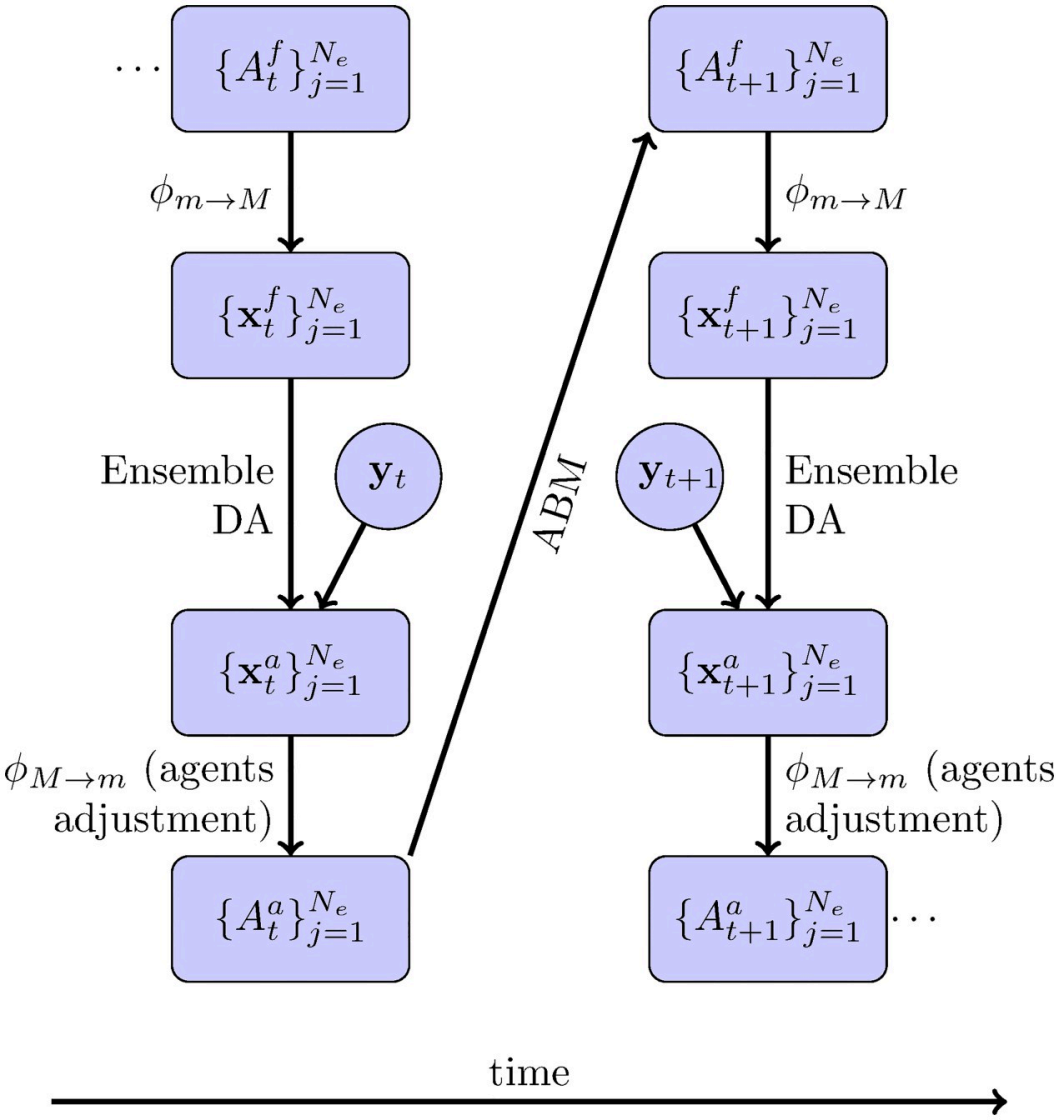
Ensemble based DA for ABMs.

At the cycle at time *t*, let us suppose we have an ensemble of micro-states ${\left\{A_{t}^{f(j)}\right\}}_{j=1}^{N_{p}}$ representing *N*_*p*_ forecasts at time *t*. Each ensemble member $A_{t}^{f(j)}$ represents an agent population, where *f* stands for forecast. We can get a state forecast by applying *ϕ* and get state variables ${\left\{x_{t}^{f(j)}\right\}}_{j=1}^{N_{p}}$. Using this forecast and the observations **y**_*t*_, we can obtain a sample of the posterior distribution, ${\left\{x_{t}^{a(j)}\right\}}_{j=1}^{N_{p}}$, so-called analysis states, through DA (the *a* supraindex stands for analysis). The next usual step in DA would be to predict the state at time *t* + 1 by evolving ${\left\{x_{t}^{a(j)}\right\}}_{j=1}^{N_{p}}$ into ${\left\{x_{t+1}^{f(j)}\right\}}_{j=1}^{N_{p}}$. The ABM cannot directly evolve the state variables **x**_*t*_ into **x**_*t*+1_ but rather it can evolve *A*_*t*_ into *A*_*t*+1_. Because of this, we need an agent representation $A_{t}^{a(j)}$ of the analysis macro-state variables $x_{t}^{a(j)}$. This agent set is then used to evolve forward in time and get a forecasted population of agents for time *t* + 1, $A_{t+1}^{f(j)}$. In this work, we propose two methods to adjust the forecasted agent population $A_{t}^{f(j)}$ to be consistent with the analysis state variables $x_{t}^{a(j)}$. With this adjustment, we get the analysis representation of the agent population $A_{t}^{a(j)}$ which we can now evolve forward with the agent-based model and get the desired forecasted agent population $A_{t+1}^{f(j)}$ completing the forecast-analysis sequence. This methodology is summarized in Fig 2.

### Agents adjustment

The main issue with the DA cycle is the macro-to-micro map *ϕ*_*M*→*m*_. For each *j* = 1, …, *N*_*p*_ we have a mismatch between $x_{t}^{f(j)}=\phi_{m\to M}\left(A_{t}^{f(j)}\right)$ and $x_{t}^{a(j)}$. Our approach to get the filtered ensemble of agent populations ${\left\{A_{t}^{a(j)}\right\}}_{j=1}^{N_{e}}$ is to use the forecasted state of agents ${\left\{A^{f(j)}\right\}}_{j=1}^{N_{e}}$ and change the least possible number of labels to match the analysis macro-state variables $x_{t}^{a(j)}$. This is inspired by the idea that the agents representing the analysis are a correction of the agents representing the forecast. The better the forecast is, the fewer agents have to be changed. However, the inner structure of agents can be very complex, with many other different attributes aside from the epidemiological status requiring an adjustment. Whether or not it is possible to adjust these attributes realistically will depend on the ABM and on how much of the agent’s inner structure is represented by the macro-state variables.

In order to match the agent state to the filtered state, in the first proposed correction method, the epidemiological category which lack agents will take the necessary number of agents from the categories which show an excess. The agents are selected randomly and because of this the method is called *randomized redistribution*. This is achieved through a change of labels and the procedure is repeated independently for every location. Furthermore, not only the labels have to be changed, but other attributes may need to be changed as well. For instance, every agent in either *E*, *I*_*M*_, *I*_*S*_ or *H* has a time counter which counts the remaining time in the current category and, when it expires, it indicates that the agent has to leave its current class and enter the next one. These time counters are originally sampled from Gamma distributions, as explained before. Then, when an agent is changed in the adjustment to one of these categories its counter has to be reset. Our choice for this is to sample this counter from the current distribution of the counters of agents already in this category. We make this choice in order to be as least intrusive as possible with the agent populations. Although this implementation is particular to this problem (the proposed epiABM model), a similar approach can be taken in general as long as we have some prior knowledge on the distribution of values of particular attributes. An advantage of this method is that the amount of agents that change category is the minimum possible.

We implement a second method to adjust the agents which does not necessarily minimize the amount of changes needed but it aims at preserving the history of each individual agent. The choice of the agents which change epidemiological category attempts to select the most likely agents to suffer a change. Changes are made only between adjacent health classes in the epiABM progression chain, starting from the latest categories (*R* and *D*) and ending in the first class (*S*). Also, the selection of the agents to be changed is not random. Considering the time counter of each agent in its category, if a correction needs to be done in the direction of the flow of the diagram, the agents that spent more days in their current category will be transferred to the next. Conversely, if a change is required in the opposite direction, the agents with fewer days in their category are selected to return to the previous one. The idea for this criterion is to preserve individual trajectories on the flow of the infection dynamics. For the transitions between susceptible and exposed agents, the time criterion in the susceptible class does not apply. In this case, the criterion to move agents from the susceptible to exposed categories is the number of risky contacts it had (i.e. the number of contacts it had with infectious agents, which do not always result in an infection). In the opposite direction, to move agents from the exposed category to the susceptible we keep the criterion based on the days it spent in the category. Whenever an agent changes its category, the counter is reset with new values sampled from the corresponding attributes of the population where it has been reassigned. The procedure is applied to each location separately. The method is named *backward cascade redistribution*. The downside for this method is that when we select agents with too few or too many days in their categories to change their status we are cutting off the tails of the distribution of the time spent in each epidemiological class.

## Experiments and results

We conducted a set of experiments using the epiABM coupled with the proposed methodology based on the EnKF. First, we use synthetic observations to evaluate the overall performance of the system, in which the true state is generated with the ABM with a set of prescribed parameters. Then, we use data from the reported cases in CABA, Argentina.

For each neighborhood, we have 7 macro-state variables which correspond to the variables (*S*, *E*, *I*_*M*_, *I*_*S*_, *H*, *R*, *D*). So if we consider *N*_*loc*_ neighborhoods, the dimension of the state will be *N*_*x*_ × *N*_*loc*_ without counting the possible parameters which augment the state for parameter estimation. Default parameter values are listed in Table 1. If there is no explicit mention of these, it means the value is from the table.

The EnKF is rather robust to noisy observations, indirect information (e.g., nonlinear observational operator), and partial observations (incomplete state). In the experiments, we assumed the cumulative confirmed cases for each location is observed, which is defined as the sum of *I*_*M*_, *I*_*S*_, *H*, *R*, *D* for each neighborhood. The cumulative number of deaths per location is also observed. The error in the observations is considered to be zero-mean Gaussian noise with variance proportional to the observed quantities themselves. The coefficients of proportionality used are named *κ*_*C*_ and *κ*_*D*_, respectively. The possibility of asymptomatic undocumented cases in the cumulative confirmed cases is considered in one of the synthetic experiments.

There are several versions of the EnKF. In this work, because the state space has relatively small dimensions, we use a classic implementation called EnKF with perturbed observations [43]. Because of sampling errors and unrepresented model errors in the prediction state ensemble, the prediction sample covariance is underestimated, so that the EnKF ensemble usually has a tendency to collapse, i.e. underestimation feedback between prediction uncertainty and posterior uncertainty. To mitigate this effect, the common methodologies are multiplicative covariance inflation [44, 45] or additive Gaussian noise in the state variables of the ensemble member updates, i.e. additive inflation. After some preliminary experiments, we found that the intrinsic stochasticity of the epiABM results in forecasts with enough spread so that covariance inflation is not required. In other words, the stochasticity in the ABM gives a reasonable representation of the model error. The initial variability of agents populations is given by random sampling at the initialization of agents attributes.

A sensitivity analysis to determine a suitable ensemble size was conducted for a synthetic experiment and it showed that 50 members are enough to obtain an optimal root mean square error (RMSE). On the other hand the variance is slightly underdetermined in the 50-member experiment with respect to larger ensemble sizes. We chose a 100-member ensemble for the synthetic observations experiments as a robust choice giving optimal RMSE and accurate variance representation. For the case of the real data experiment, the RMSE is not possible to be calculated (because the true state is unknown) so that the sensitivity analysis is not possible. We use a 400 ensemble size in the real data experiment because the state space is about four times larger than in the synthetic experiment (due to the number of neighborhoods). In both cases, the ensemble sizes we chose are big enough that using larger ensembles would not yield detectable changes in the results.

### Synthetic observations

In these experiments, we produce synthetic observations using the epiABM and then estimate state variables using the EnKF. Observations are produced using Eqs 1 and 2. Since the true state which produced the observations is available, we can examine the technique’s performance. In most experiments the model used to simulate the observations is the same as the one used by the EnKF. However, in some experiments, we consider unknown parameters and even some model misspecification.

For these experiments, we use four neighborhoods, *N*_*loc*_ = 4. The contact matrix is set to:

$$\begin{array}{c} C=(\begin{array}{cccc} 0.43 & 0.14 & 0.14 & 0.29\\ 0.14 & 0.43 & 0.14 & 0.29\\ 0.14 & 0.14 & 0.43 & 0.29\\ 0.14 & 0.14 & 0.14 & 0.57 \end{array}). \end{array}$$ (3)

We consider that agents have more contacts within their own neighborhoods which explains the larger values in the diagonal elements. Also, one of the neighborhoods (the one with largest index) is more concurred by all the agents, representing a city center: this explains the larger values in the last column.

#### Time varying number of contacts

In this experiment, the number of contacts an agent has in a day, which is encoded in λ, is considered to decrease linearly in time in the true simulation. This parameter is assumed unknown for the method and is estimated through state augmentation. The simulation uses 3 ⋅ 10^4^ agents and the observational error coefficients are set to *κ*_*C*_ = 1 ⋅ 10^−5^ *N*_*agents*_/*N*_*loc*_ and *κ*_*D*_ = 1 ⋅ 10^−6^ *N*_*agents*_/*N*_*loc*_.

**Fig 3:**
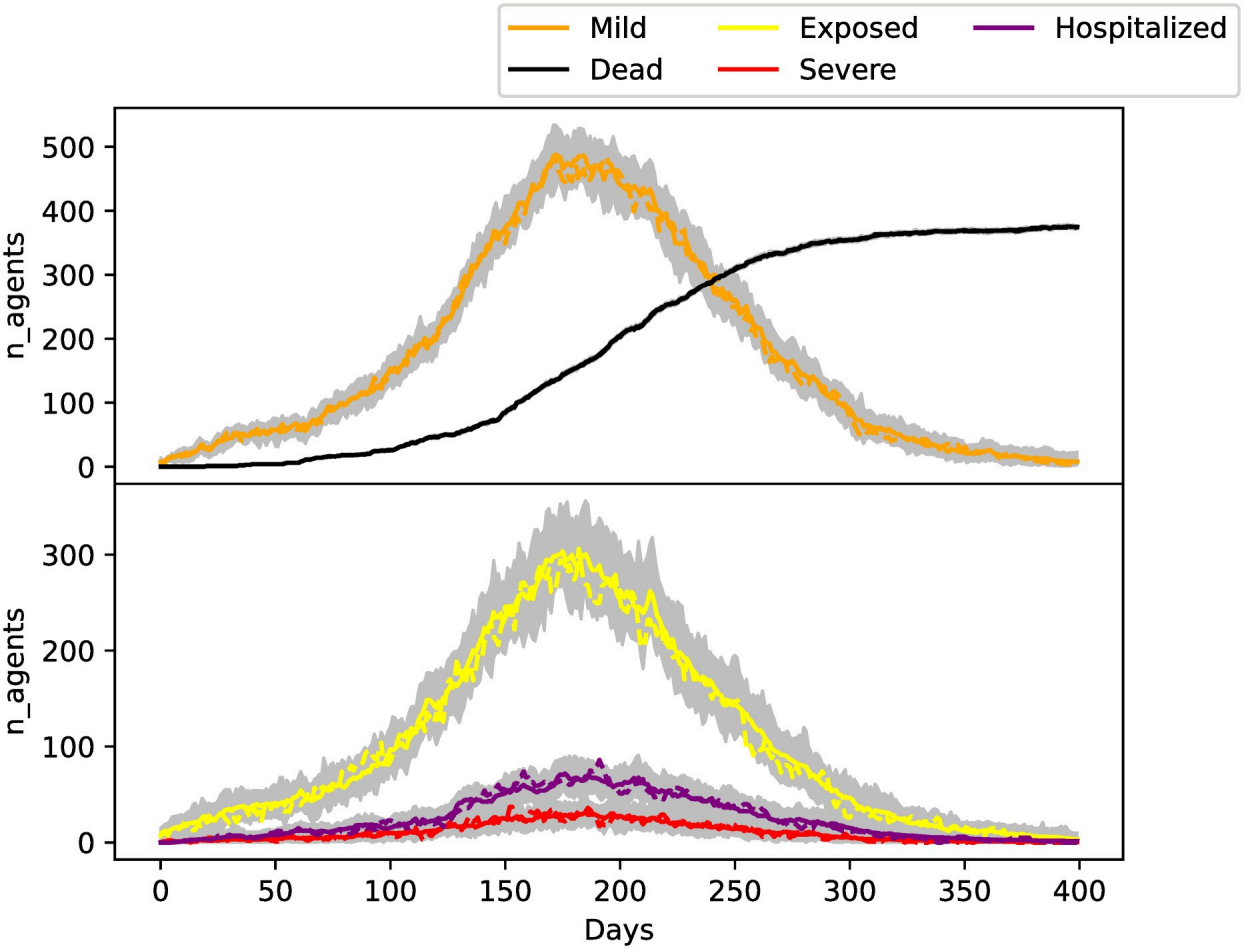
Total number of agents in different epidemiological categories summed over every neighborhood. Grey lines indicate ensemble members and colored solid lines their mean. True values are indicated with dashed lines.

**Fig 4:**
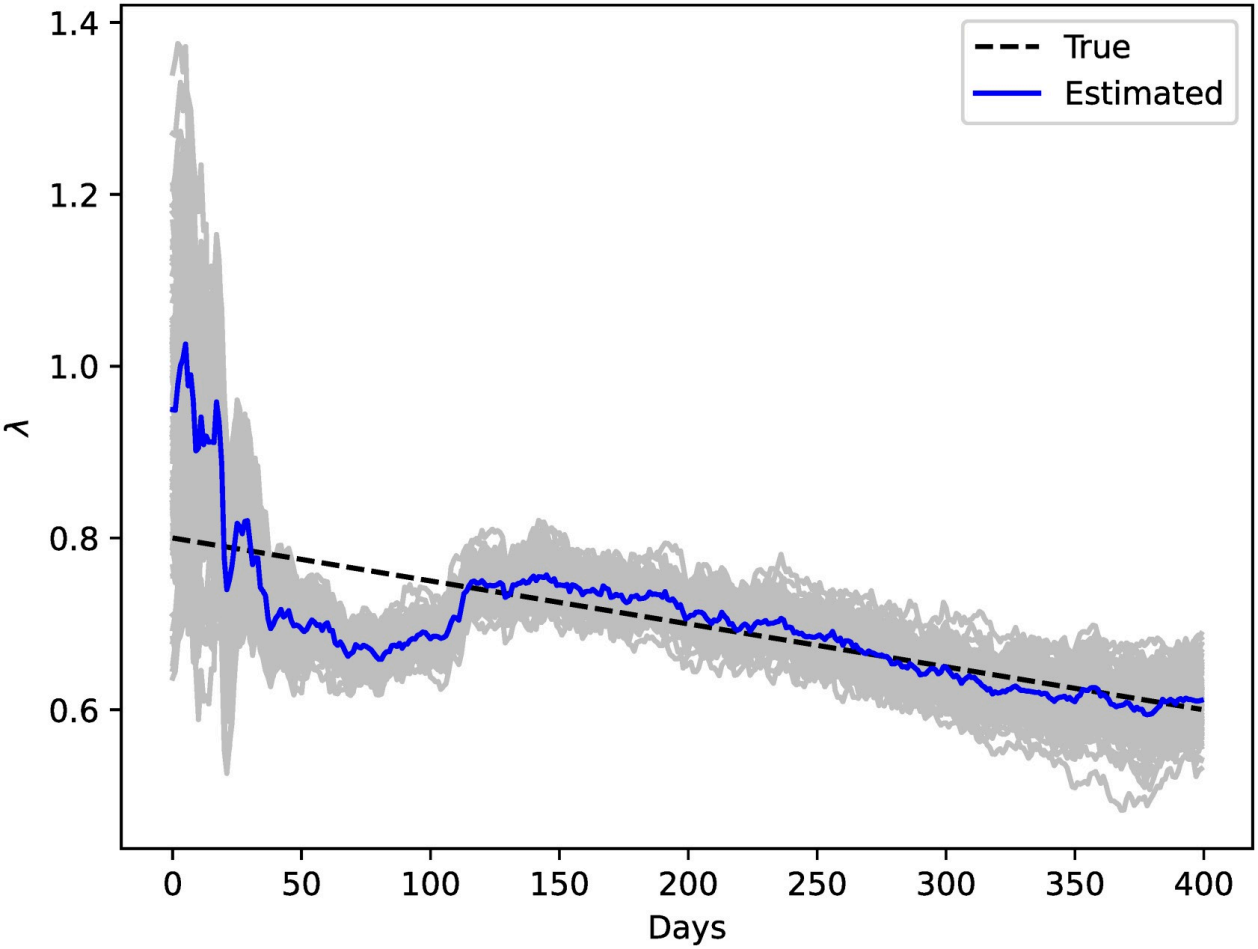
Estimations of λ. The estimated values as a function of time are shown in solid blue line. Grey lines indicate ensemble members. The true evolution is drawn with a dashed line.

Fig 3 shows the true trajectories of the state variables and the estimates produced by the EnKF. The filtered ensemble variables are able to correctly estimate the true state variables. The cumulative deaths given by the ensemble members representing the posterior density has very little variance. This is because this variable is directly observed and with a relatively small observational error. In contrast, the other state variables are not observed: they are estimated through the correlations with the observed variables. Fig 4 shows the true value of λ over time and the estimates produced by the EnKF with randomized agent redistribution. The results are shown for the randomized adjustment method but similar results were obtained for the backward cascade method. The estimated values can capture the change of the parameter in time after some initial assimilation spin-up time. The estimated parameter values are closer to the true value at the epidemic peak. At around 300 days, the variance of the λ estimations starts to grow. This is because observations are more informative on λ when the number of new infections is higher. After 300 days, the number of active cases is small; correspondingly, the parameter uncertainty increases at those times.

The initial prior mean parameters and state variables are chosen different from the true values because they are assumed to be unknown. Besides, the variance of the initial ensemble is chosen to be large as is standard in DA in order to better explore the parameter space. Initial parameter correlations are set to 0. As the observations are assimilated the ensemble starts to improve the prior state and it slowly synchronizes with the true value. Correlations between variables and variable-parameter correlations are expected to be captured after a number of cycles. As the parameter-observed variable covariances become higher, the ensemble parameter estimates gain more accuracy, and so the ensemble parameter spread begins to shrink. This initial spin-up is a typical behavior of DA. In general, a longer assimilation spin-up is expected when estimating parameters with data augmentation, because parameters are not (directly) observed.

**Fig 5:**
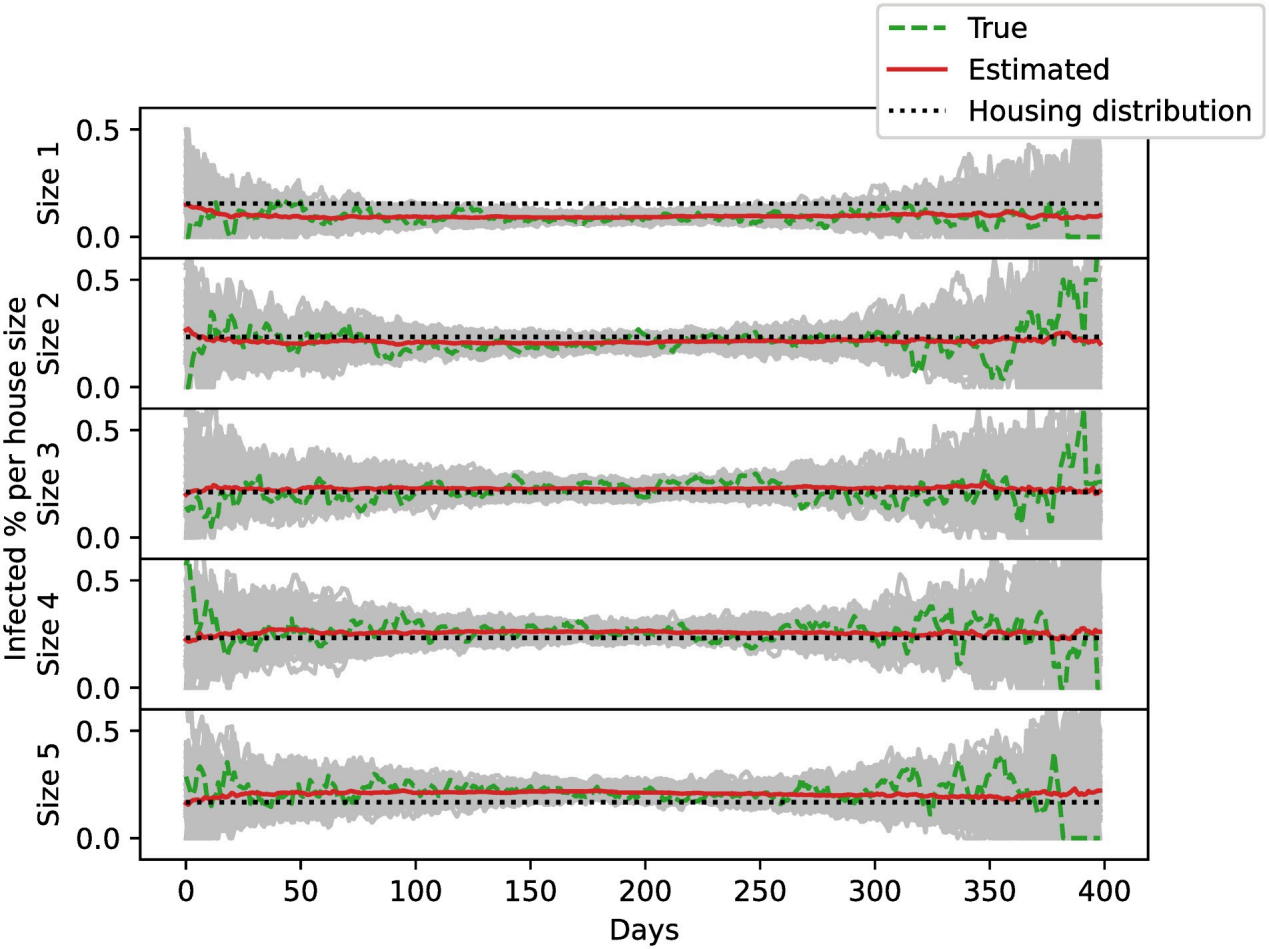
Relative amount of infected agents per house size as a function of time. Ensemble members plotted with gray and their mean with red. The true values are represented with green dashed lines. Dotted black lines indicate the distribution of agents in different house sizes regardless of their disease status.

The structure given by the distribution of agents in houses is not directly informed on by the observations. However, the model keeps track of how the infections were distributed in different types of houses. The house sizes we considered range from a minimum of a single agent per house to a maximum of five. At each simulation day, we calculated what proportion of the total infected corresponds to each household size. Fig 5 shows the evolution of the proportion of infections which occurs at each house type. When there are fewer infections, the estimates have higher variance and become more accurate and with less dispersion at the time of the epidemic peak. The proportion of infections in larger houses is greater at the beginning of the epidemic and lower by the end. We can compare the proportion of infected in each house type with the number of agents residing in them regardless of their disease status. In smaller houses, the estimated proportion of infections is less than the proportion of agents residing in that particular house types. The opposite effect occurs with larger houses. This is due to the fact that, because of domestic contacts, the infections spread faster in houses with more members.

#### Assessment of microscale tracking

We conducted an experiment to evaluate the proportion of agents with matching epidemiological states (between true and estimated agent populations) at different levels of aggregation. The aim of this experiment is to compare the evolution of the agent micro-states between the true agent population that produced the (synthetic) observations and the agent micro-states obtained in the ensemble members of the filter. For this comparison, we count the number of agents with the same epidemiological status, but this count is carried out by considering different groupings for the agents. The first metric (agent_id) considers agents id per id. The second metric (house_id) groups agents according to their house id so we count the number of matches house per house. The third metric (household_type) groups by house size and the fourth (loc_household_type) by house size and location. To count the number of matches in the metrics which do not distinguish agents by id we simply count the number of agents in each population that are in the same category.

As in previous experiments, we use *N*_*loc*_ = 4 locations but for each different location *i*, we consider a different λ_*i*_. Specifically, we set (λ_1_, λ_2_, λ_3_, λ_4_) = (1.0, 0.8, 0.9, 0.7). We use populations of 5 ⋅ 10^3^ agents and the observational error coefficients are set to *κ*_*C*_ = 1 ⋅ 10^−4^ *N*_*agents*_/*N*_*loc*_ and *κ*_*D*_ = 1 ⋅ 10^−5^ *N*_*agents*_/*N*_*loc*_.

The starting configuration for the agents in the true run and the ensemble members is the same, so the matching proportion at the initial timestep is 100% for every metric. This amounts to have a Delta distribution for every agent population. To evaluate the impact of the assimilation with respect to control simulations, we produce 100 trajectories with the model without any DA. These will yield different results to the true trajectory because of the model’s stochasticity, so it is useful reference to compare the metrics.

**Fig 6:**
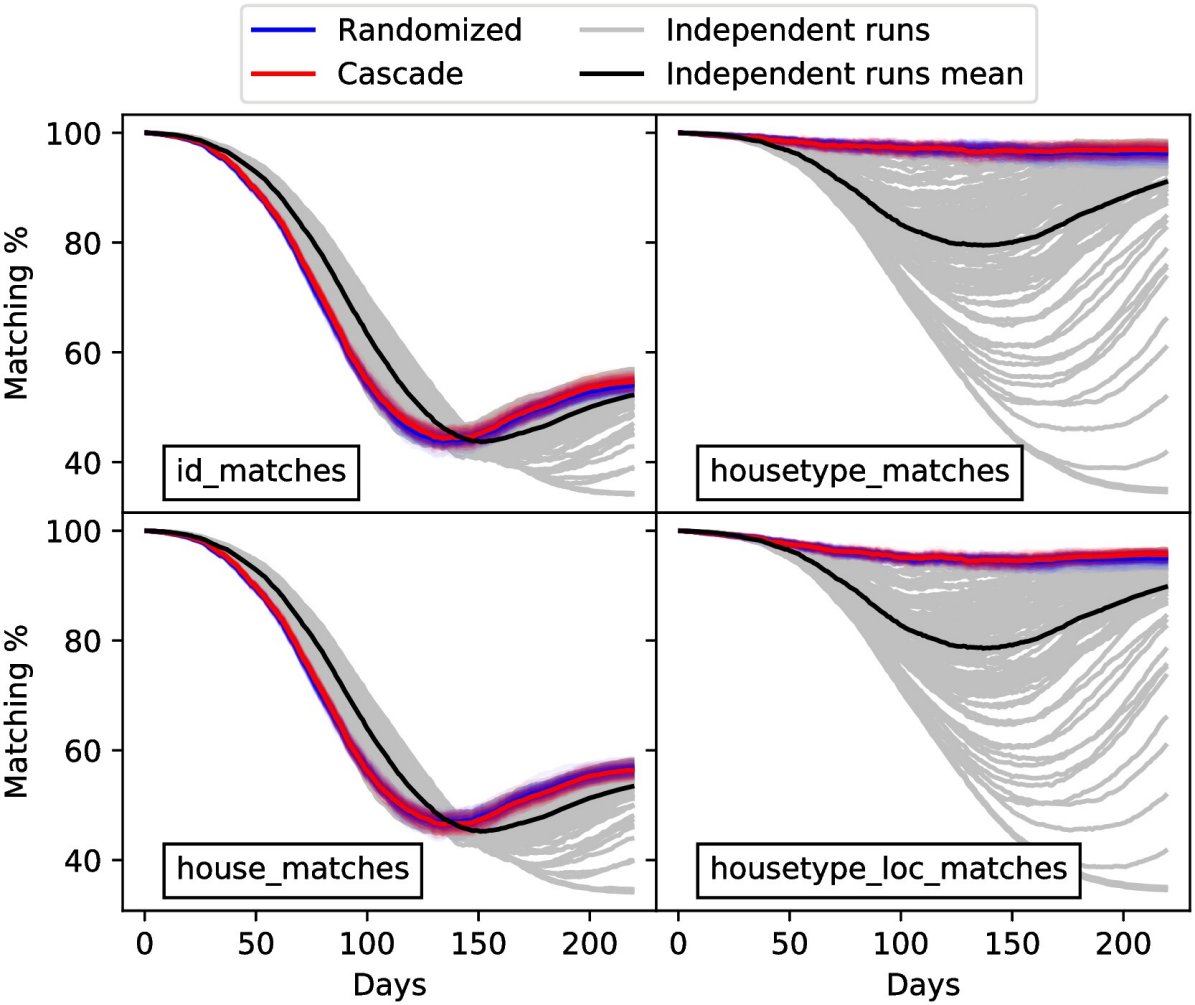
Evolution of matching metrics over time. The evolution of the id_matches, housetype_matches, house_matches and housetype_loc_matches to the true state are shown from left to right and top to bottom panels. For every panel, the gray lines represent the metrics for the control simulations and their mean is drawn with a black line. Red (blue) transparent lines correspond to EnKF estimation with the randomized (cascade) agent adjustment method. Semitransparent lines represent the ensemble members while their mean is drawn with a solid line of the corresponding color.

Fig 6 shows the metrics obtained with the assimilation system for both agent adjustment methods and for the control simulations. Each panel shows one of the different metrics. The randomized and cascade methods are very similar in all the cases. The metrics for control simulations are highly spread out, while for the ensemble members of the EnKF they are constrained. For the agent_id and house_id metrics (these are the metrics which inform on a more microscopic scale) the matching percentage drops, moderately recovers and then stabilizes both for the EnKF and the control simulations. The control simulations are slightly more consistent with the true run that the EnKF runs at the first stage when all metrics decrease. This is likely so because the agent adjustment method by the EnKF has to modify the agent populations in order to match the macro scale while the control simulations maintain the structure of the initial true agent configuration less disrupted.

At the house type scale, the EnKF maintains a high matching proportion (above 90% for both agent adjustment methods). The control simulations exhibit a large variability. The loc_household_type metric yields similar results. Some control simulations mismatch the epidemic peak of the true run. Thus, a decrease of the metrics is expectable for these simulations. The matching proportion recovers at the end of the simulation because the size of the epidemic is similar for the control simulations and the true run, and most agents will be either recovered or susceptible. For the EnKF estimations, a good level of matching is obtained, which is explained because the EnKF is able to track the true state. It is worth noting that observations of the state are classified by location but are not explicitly informative on different house sizes. However, the matching percentage of epidemiological cases at different types of houses is quite high. The proportion of matches when considering agents or houses ids is similar to not using any DA. This is likely because the specific ids of houses and agents have no particular role in the infection dynamics. On the other hand, the house type does have a role in the propagation dynamics and so it is better captured by the EnKF.

#### Estimation of undetected infections

**Fig 7:**
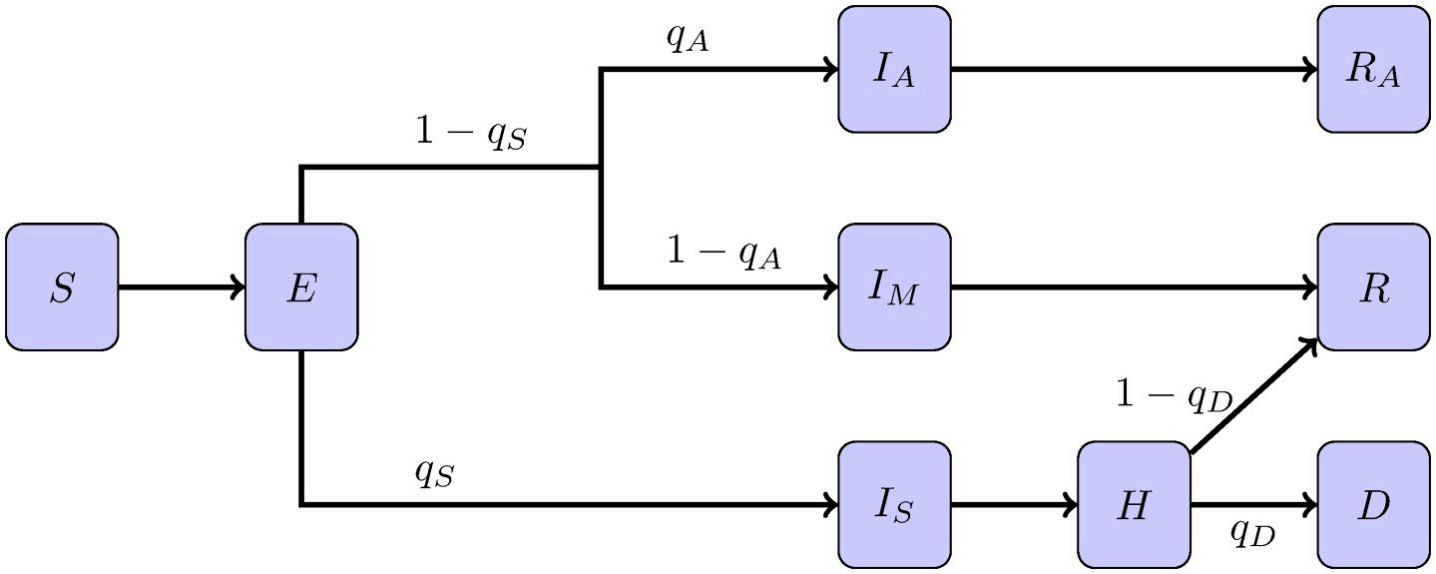
Diagram for epiABM with categories to account for unreported cases.

Data on COVID-19 infections is expected to be underreported because of mild or asymptomatic cases. In order to evaluate such scenario, we consider that mild infections have a chance *q*_*A*_ of being asymptomatic, and they are not reported in the data. We use the same setup as in the previous experiment but considering a fixed value λ = 0.8 and introducing *q*_*A*_ into the augmented state. We change the agent-based model incorporating new epidemiological categories in order to account for this. We incorporate the asymptomatic and asymptomatic recovered epidemiological states. The diagram for the disease development through the epidemiological classes is represented in Fig 7 where *I*_*A*_ and *R*_*A*_ stand for infected and recovered asymptomatic, respectively. We note that this scenario could be also represented by assuming unknown coefficients of the observational operator $H_{t}$ and estimating them through state augmentation.

The observed variables are the cumulative confirmed cases (sum of *I*_*M*_, *I*_*S*_, *H*, *R*, *D*) and deaths (*D*) per neighborhood. We introduce a new observational variable to the state called global positivity which consists of the proportion of the population which is infected. Asymptomatic cases will be ignored by every observed variable except for the global positivity. To produce observations from this variable in the synthetic experiment, we simulate data gathered from a randomized COVID-19 testing strategy. At each simulation day, we select a random set of agents and test them. Tests will be positive when the agent is in either *I*_*M*_, *I*_*S*_, *I*_*A*_, or *H*. The tests are not disaggregated by location, so they give an idea of the global circulation of the virus. The error for the observations of the global positivity will be sampling error. We assume 1% of the population is tested every day. In practice, indirect proxies such as test results of plausible cases (which are not from random samples) could be used to infer global positivity.

For this experiment, we use 3 ⋅ 10^4^ agents and observational error coefficients *κ*_*C*_ = 1 ⋅ 10^−5^ *N*_*agents*_/*N*_*loc*_ and *κ*_*D*_ = 1 ⋅ 10^−6^ *N*_*agents*_/*N*_*loc*_. We also consider a fixed value λ = 0.8. The asymptomatic rate *q*_*A*_ (which here also accounts for undocumented cases) is estimated by augmenting the state with this parameter. The rest of the configuration is similar to the first synthetic experiment presented. The asymptomatic rate is expected to be estimated with the EnKF via correlations with the global positivity.

**Fig 8:**
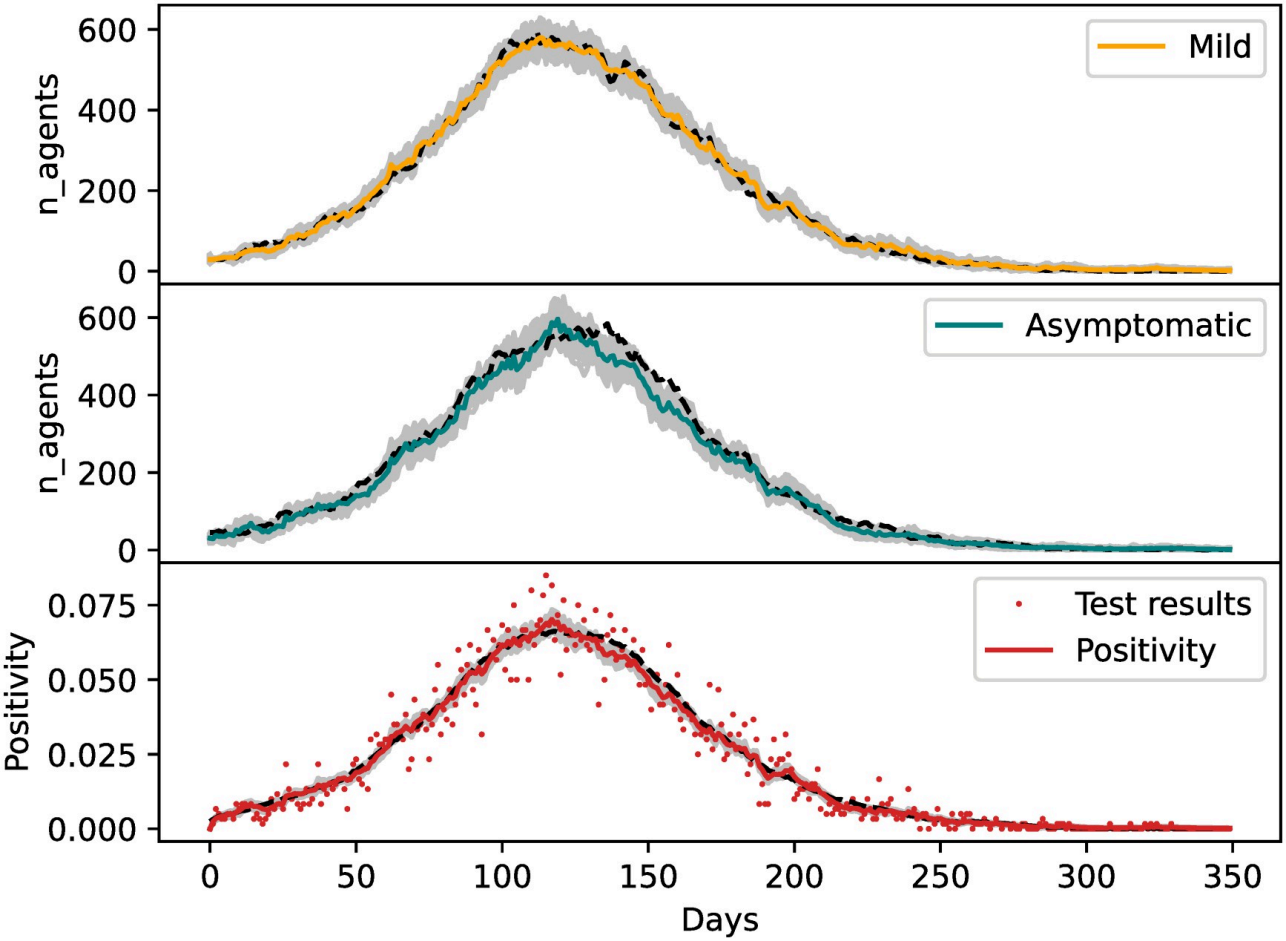
Estimations of active reported mild cases, unreported cases and positivity. Top panel: evolution of reported active mild cases. Orange solid line is the ensemble mean. Middle panel: Unreported active cases. Teal solid line is the ensemble mean. Bottom panel: Positivity (percentage of agents that would test positive). Red solid line is the ensemble mean. Dots correspond to the testing data generated with the true run. For every panel, gray lines indicate ensemble members and dashed lines the true value of the corresponding variable.

**Fig 9:**
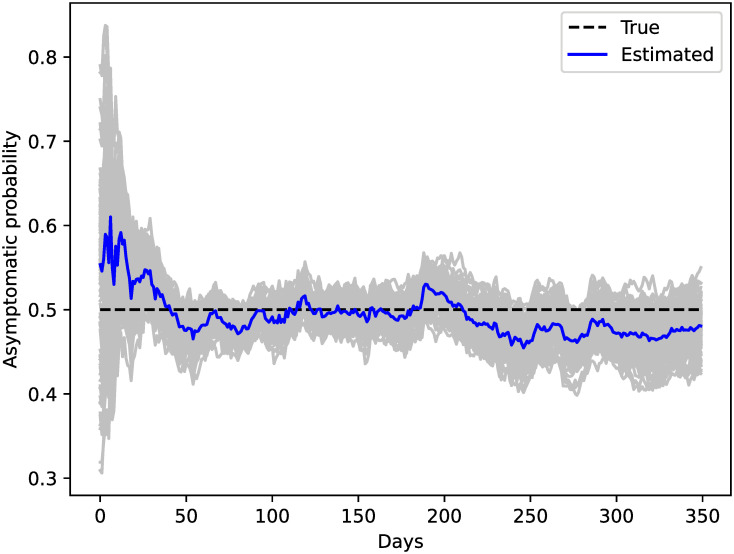
Estimations of the asymptomatic rate. The mean of estimation of *q*_*A*_ as a function of time is shown with a solid blue line, ensemble members with gray lines, and the true parameter value with a black dashed line.

Fig 8 shows the evolution of the number of mild infected agents, the asymptomatic, and the global positivity alongside tests’ results. The overall behavior of the asymptomatic agents is rather well captured by the EnKF but, as expected, the estimations are more accurate for the mildly infected than for the asymptomatic cases. This is because the asymptomatic are only informed on by test results while the symptomatic cases are also informed on by the cumulative confirmed cases. The mismatch between the asymptomatic with their true value is correlated with the mismatch between the true and estimated global positivity. This suggests that the more we know about the general circulation of the virus, the better we can infer the underreported cases. Fig 9 shows the estimation of the asymptomatic probability which gives noisy estimates around the true value of *q*_*A*_ which is 0.5. In the first cycles, we can see an assimilation spin-up with a strong reduction of the variance (similar to Fig 4). Furthermore, when the positivity is low at the start and end of the epidemic, the estimations of *q*_*A*_ do not synchronize well with the true value (because observations are less informative) and the uncertainty grows, i.e. the ensemble shows more spread. Because the global positivity is correlated with the asymptomatic infected category, *I*_*A*_, the system can use these correlations to give an estimate of *q*_*A*_.

#### Model error experiment

In order to evaluate whether the inference system is robust enough to account for a misspecification in the agent model setup, we conducted an experiment in which the observations and the EnKF predictions are obtained through different agent-based model configurations. To generate the synthetic observations, the number of daily contacts is sampled from a geometric distribution with parameter *p* = 0.5. On the other hand, the model in the assimilation uses a Poisson distribution as in previous experiments. Additionally, we use different household type distributions: the true simulation is conducted with *p*_*H*_ = (0.33, 0.27, 0.2, 0.13, 0.07) for which we have more households with few agents and less households with more agents, which is an expectable situation in real life (close to those used in the rest of the experiments which represents the CABA house distribution). For the EnKF runs we use this distribution but we also repeat the experiment with *p*_*H*_ = (0.2, 0.2, 0.2, 0.2, 0.2) (we call this uniform housing) and *p*_*H*_ = (0.07, 0.13, 0.2, 0.27, 0.33) for which we have more larger households and less households with few people (we call this distribution unbalanced housing). Different configurations for household distributions will yield different propagation dynamics due to variations in contact structures [46].

The parameter λ is assumed unknown and we estimate it through state augmentation. We use 3 ⋅ 10^4^ agents and observational error coefficients *κ*_*C*_ = 1 ⋅ 10^−5^ *N*_*agents*_/*N*_*loc*_ and *κ*_*D*_ = 1 ⋅ 10^−6^ *N*_*agents*_/*N*_*loc*_.

**Fig 10:**
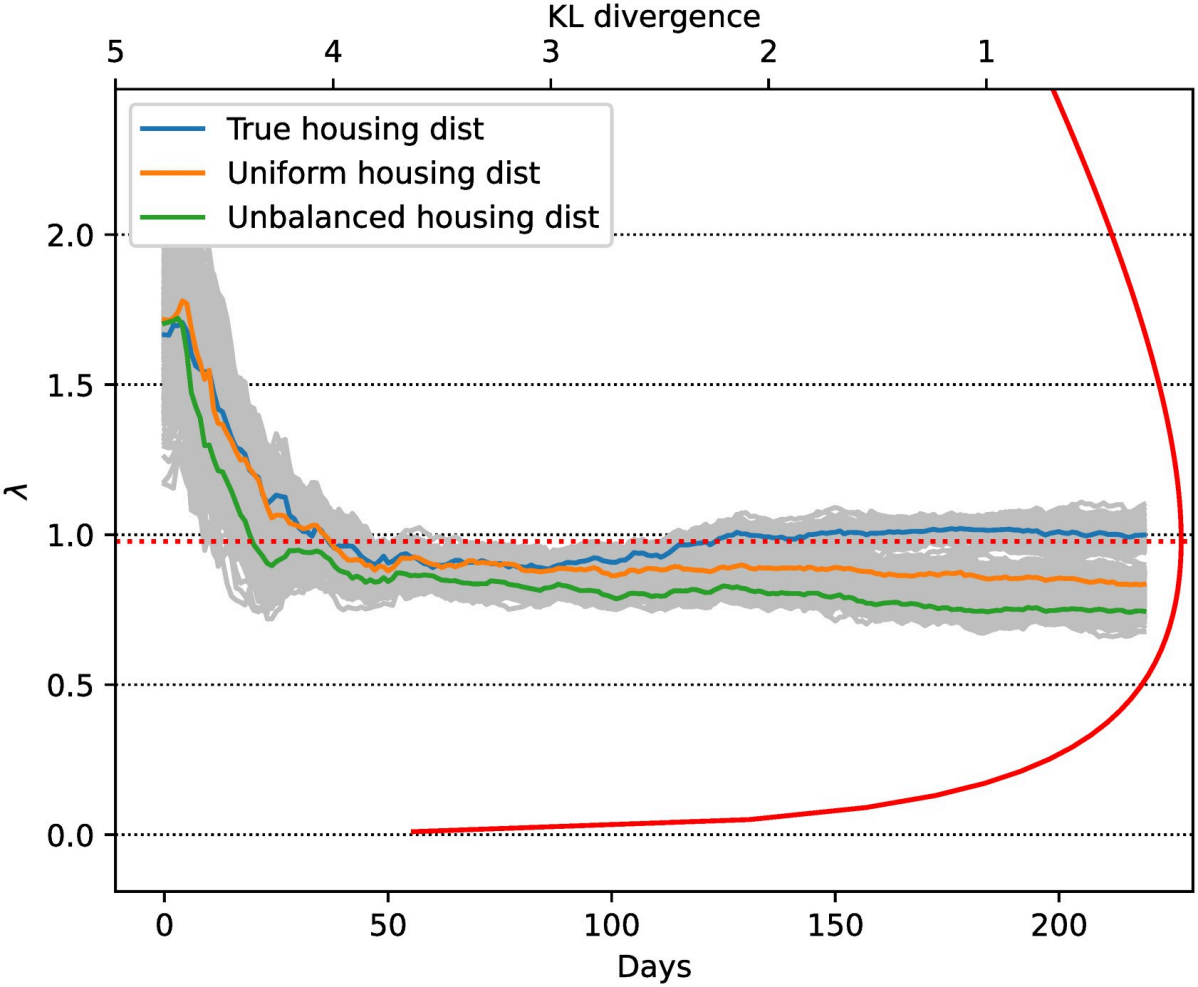
Estimations of λ for different housing scenarios. The mean λ estimates for the different housing distributions are shown with solid colored lines. Ensemble member estimates represented with gray lines. The solid red line indicates the KL divergence between a Poisson distribution and the true geometric distribution used. The dotted red line indicates the minimum of the KL curve.

Fig 10 exhibits the λ estimates as a function of time for each of the three different housing scenarios. In order to compare how the estimated distribution performs, we compute the Kullback-Leibler divergence between a Poisson with and the true geometric distribution as a function of the Poisson parameter λ. This encodes how much information is lost when we use the estimated Poisson distribution instead of the geometric. Fig 10 also shows the Kullback-Leibler divergence alongside the λ estimates. These are in a region of low KL divergence with respect to the true distribution. This means that the system self-calibrates using a Poisson distribution which causes a model behavior which resembles model’s characteristics when a geometric distribution is used. The best estimate in terms of KL divergence is for the experiment which uses the true housing distribution, which is expectable. The uniform housing distribution has more agents living in larger households and the unbalanced even more. This will produce an effect of faster spread of the disease in the model. The fact that the λ estimates are smaller in these cases is because the system is self-calibrating by estimating λ. A smaller value for λ compensates for the misspecification of the housing distribution. Comparatively, the disease dynamics propagate more by intra-house contacts than by external contacts in the latter.

### Ciudad Autónoma de Buenos Aires data

To evaluate the system with realistic observations, we use the data on COVID-19 cases for CABA, Argentina. The data is provided by the Ministry of Health and published in https://data.buenosaires.gob.ar with daily updates. The distribution of houses and population is taken from census data also publicly available through the same website. CABA is divided into 15 neighborhoods (or communes) and epidemiological data is disaggregated according to these. We use the number of cumulative confirmed cases and deaths. The observational error variance is considered to be proportional to the observed variables. We estimate the parameter λ (which is assumed to be the same for every neighborhood) through state augmentation. To construct the contact matrix we considered that half of the casual contacts are within each commune and that the other half of the casual contacts is distributed among other communes proportionally to their population density. The number of agents used is 3 ⋅ 10^5^, and CABA has a population of around 3 ⋅ 10^6^, so we scale down the data by 10. The observational error coefficients are *κ*_*C*_ = 5 ⋅ 10^−6^ *N*_*agents*_/*N*_*loc*_ and *κ*_*D*_ = 5 ⋅ 10^−7^ *N*_*agents*_/*N*_*loc*_.

**Fig 11:**
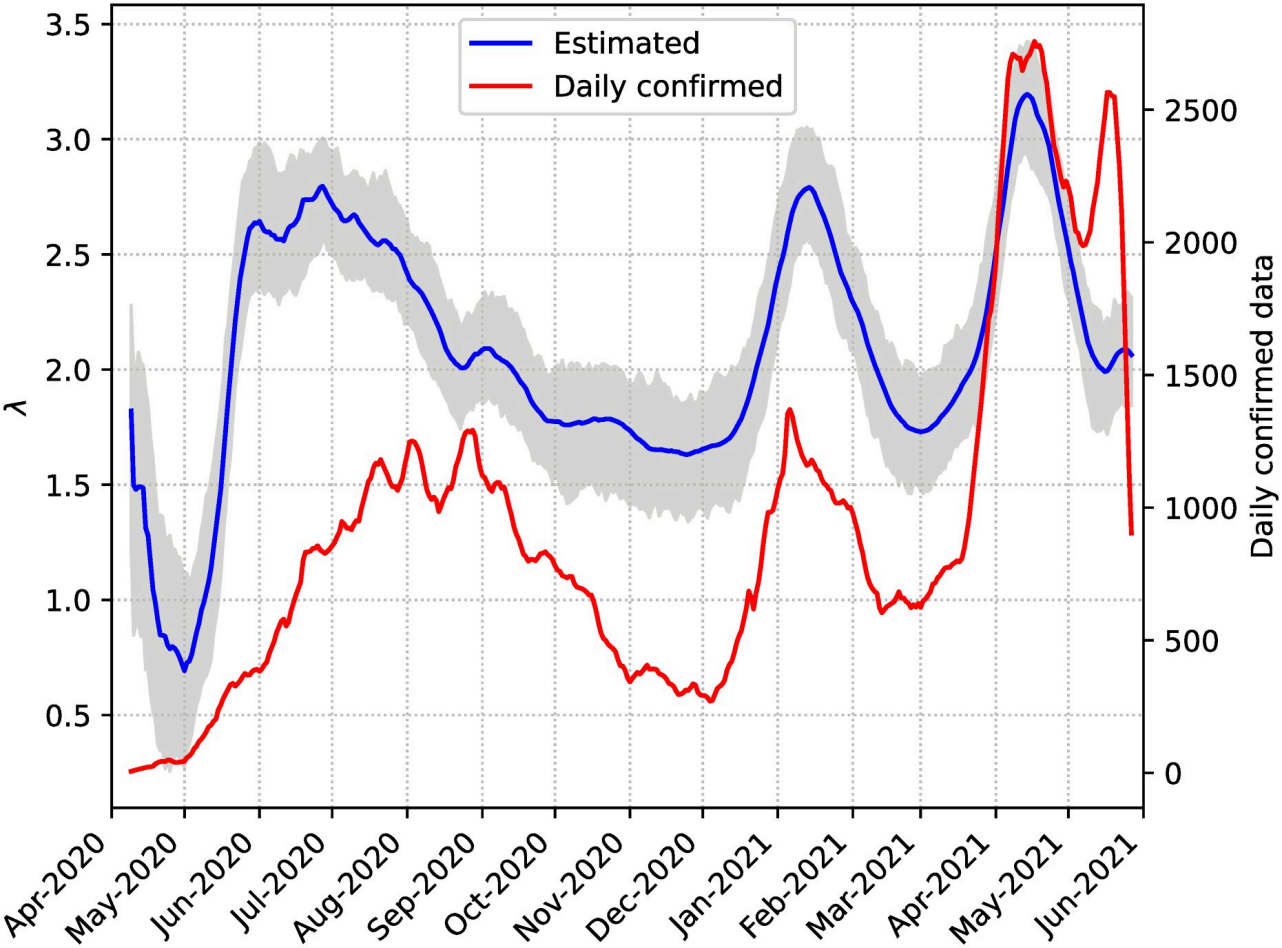
Estimations of λ. The estimated values are shown with a solid blue line and ensemble members with gray lines. The red line corresponds to the 7-day rolling average of daily confirmed cases.

Fig 11 shows the estimated λ. This parameter measures how many contacts, on average, an agent will have on a given day. We can expect this to be correlated to the number of confirmed daily cases, so we plot the data of the daily reported cases alongside the λ estimations, and indeed we can see that they follow a similar trend. This happens because *β*_*d*_ and *β*_*c*_ values remain constant throughout the simulation, so changes in the number of new cases are determined by changes in the value of λ with the corresponding time lag due to the incubation period. In the figure we show a 7-day rolling average of the daily cases but the experiment was performed on the original data without this processing.

The evolution of the estimated contact rate in Fig 11 is coherent the three epidemic waves registered in Argentina during the experimental period. There was a first period of a strong lockdown between April and May, which was then relaxed during austral winter giving place to the first wave. The second wave occurred not surprisingly in January-February which is summer holidays time in Argentina. The strongest wave is the third one, but it started fading because of, among other factors, massive vaccinations starting in May 2021.

It is important to note that of the many time-dependent processes that may affect the development of the disease spread, we only estimate λ while keeping other parameters fixed. This means that the calibration of the model to fit the data acts through λ, even though what drove the effect on the data may have another cause. For example, a decrease in cases because of the use of face-masks should be accounted for by a change in the casual contagion probability *β*_*c*_, but since we are keeping this parameter fixed, this change will be captured by λ. Although this may be inaccurate, we found that trying to estimate parameters with similar effects on data leads to overparameterization and lack of identifiability.

**Fig 12:**
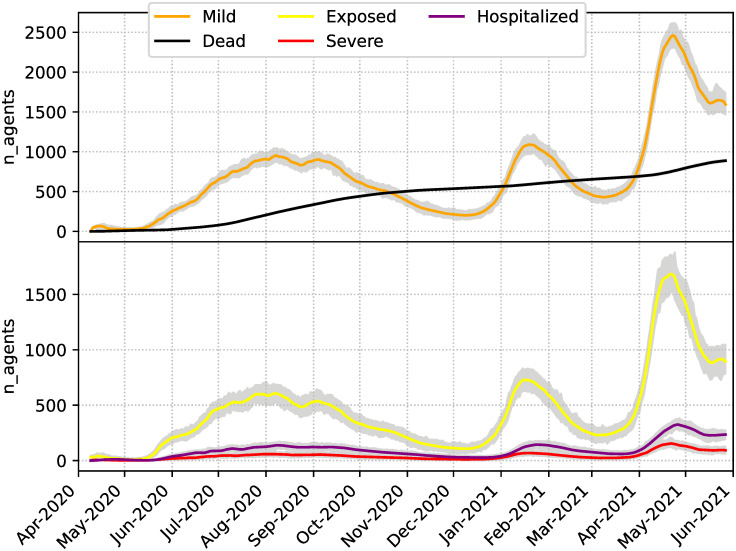
Total number of agents for different epidemiological categories. Grey lines indicate ensemble members and colored solid lines their mean.

Fig 12 shows some of the aggregated state variables of the system summed over every neighborhood. The cumulative deaths have comparatively less variance than the other variables. Like in the experiments with synthetic observations, this is because deaths are directly observed, and the other variables are only observed through the cumulative cases, which is a sum of several epidemiological states.

**Fig 13:**
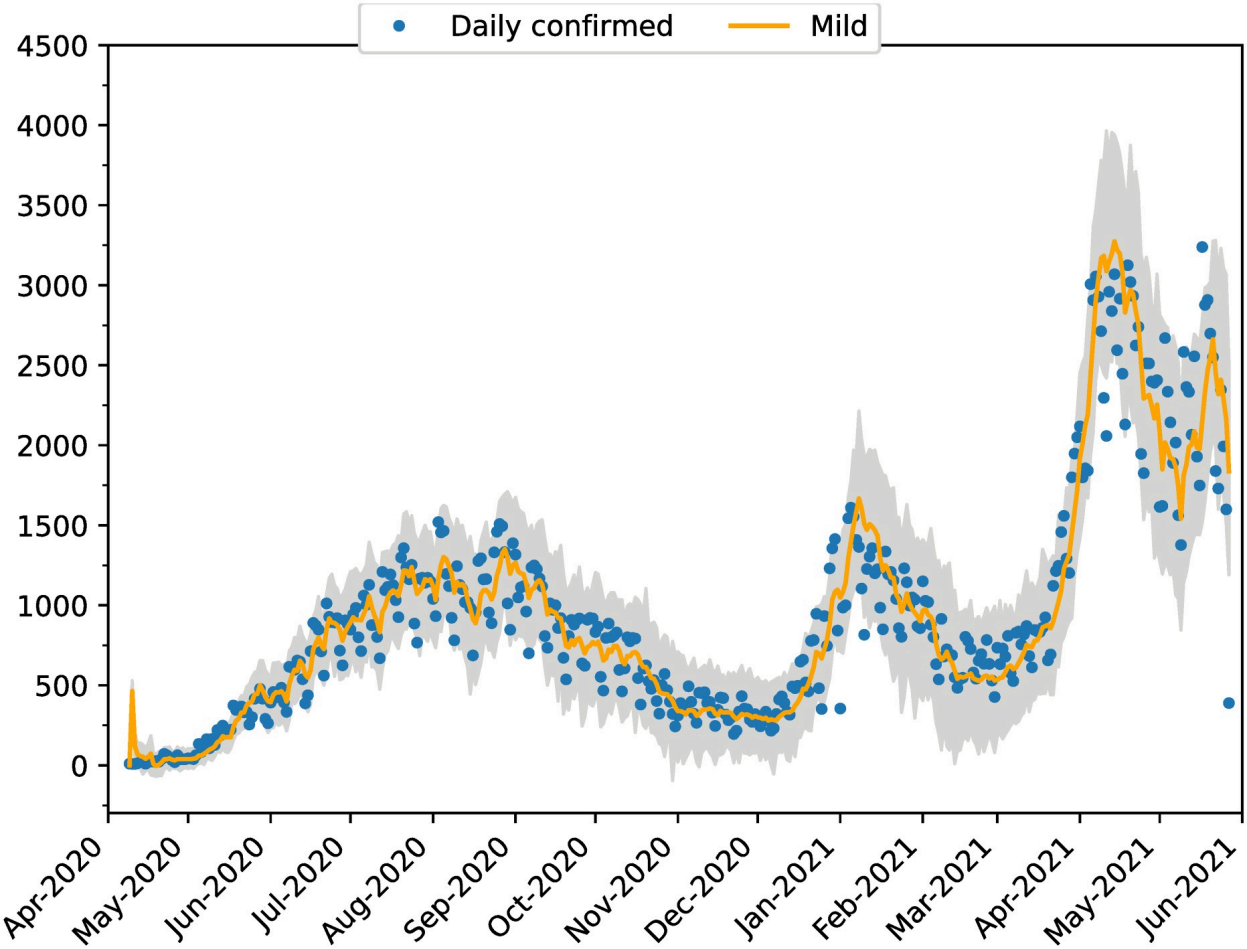
Total daily new cases. Estimated daily new cases for the whole city are shown with orange lines and the corresponding ensemble with gray. Blue dots correspond to the daily confirmed cases.

**Fig 14:**
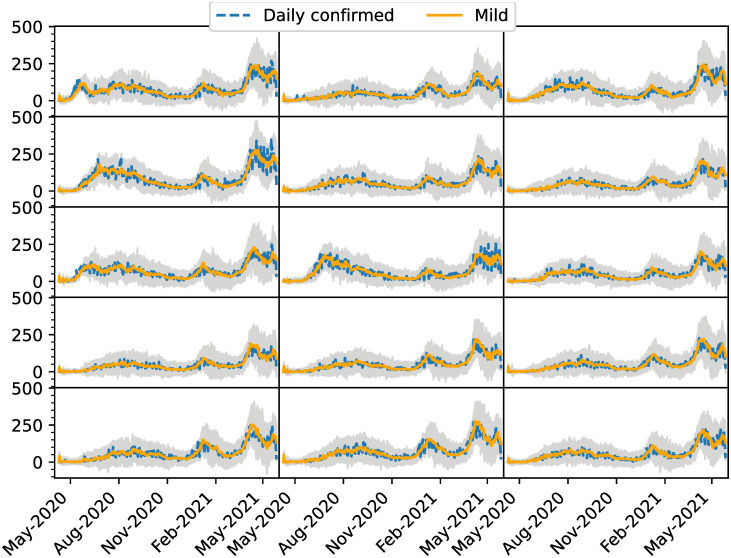
Daily new cases per commune. Each plot represents one of the 15 CABA communes. Estimated daily new cases are plotted with orange lines and the corresponding ensemble with gray lines. Blue dots correspond to the daily confirmed cases.

Fig 13 shows the estimated number of daily new cases as a function of data of confirmed new cases. The data are affected by underreporting on weekends, but the estimations tend to smooth out that effect. Fig 14 shows the same metrics but disaggregated by neighborhood and we can see that although all the neighborhoods show similar trends, some have particularities in the shape of the epidemic peaks. These are rather closely captured by the EnKF estimations.

## Discussion

With the increase of expressiveness and complexity in epidemiological ABMs, there is a current need for calibration techniques that allow to constrain parameter values and force improvements of the epidemiological state estimates. In this work we introduce and evaluate the use of DA techniques to calibrate COVID-19 ABMs using observations. DA provides a probabilistic framework to enhance model predictions and calibrate parameters by incorporating data in real time. From the data assimilation side, ABMs present some challenges since the dynamical state in ABMs is not represented by macroscopic differential equations. In ABMs the macroscopic state emerges from the microscopic dynamics of the agent population. The proposed method is designed for cases in which the only type of observed variables may be aggregated information of the agent population state. The applicability of the methodology depends on the possibility to adapt the forecasted agent populations towards the observed macroscopic state variables. We developed two different methodologies to do this, referred to as randomized redistribution and backward cascade redistribution. They both yielded similar results. We found that it is possible to track the macroscopic state variables even when the system is driven by microscopic dynamics. Also, we show that the state augmentation technique can be used to calibrate the model in a sequential manner, tracking the dynamics of a parameter which changes slowly over time. The methodology responded well to data produced by the daily monitoring of the COVID-19 pandemic in CABA. In particular, the model calibration adapts the parameterization to fit the changing behavior of the spreading of the disease.

The experiments in this work show that the EnKF is a robust Gaussian technique for epidemiological ABM data assimilation. However, many of the challenges posed by DA are inherited by the framework we present: for example, specification of model and observational errors and dealing with non Gaussianities. Alternatively to the EnKF, there is a vast variety of DA methods which could possibly be helpful to do inference alongside ABMs. Methods which do not make sequential updates but jointly assimilate data over a time window (for example, ESMDA or pMCMC) could prove useful to circumvent the need of adapting the agent populations to the macroscopic state. Among sequential methods, particle filters which rely on resampling in order to transform the forecast into the filtered ensemble, such as the bootstrap particle filter, could also be interesting to apply. The fact that the filtered ensemble is just a resample of the forecast particles means that there would be no need to readjust the agent populations.

The assimilation system shows sensitivity to the distribution of the number of households in the experiments. This is a bottom-up effect produced by the agent-based model which cannot be readily modeled with compartmental models. The coupling of the EnKF with the agent-based model may be useful, in future work, for the modeling of disease propagation in cities with vulnerable neighborhoods of developing countries, in which the proportion of households with a large number of members is much higher.

The computational cost of the methodology can quickly escalate since each ensemble member consists of a whole agent population. However, there is much room for improvement because several tasks may be parallelized (at the agent population level and also at the ensemble level). The computational cost of DA techniques themselves is usually associated with matrix inversions in high dimensions. However, that was not the case because we applied DA to the aggregated state variables, which constitute a relatively small sized state space.

In this work, all the observations are aggregated state variables, but the technique can potentially be applied for inference on the agents using data which is gathered at that micro scale. The fact that more individual-based data is collected through mobile devices is making this sort of data more accessible every day, for example, from digital contact tracing apps [47, 48]. It is expectable that the technique proposed in this work, based on the EnKF, may be able to assimilate these micro-state observed variables.
